# Genome-wide sRNA and mRNA transcriptomic profiling insights into carbapenem-resistant *Acinetobacter baumannii*

**DOI:** 10.3389/fcimb.2024.1419989

**Published:** 2024-07-30

**Authors:** Yong Wei, Xuli Xin, Jiachun Zhang, Qifeng Liao, Yan Rong, Ying Zhong, Meiying Zhao, Jianping Ma, Song He

**Affiliations:** ^1^ Department of Clinical Laboratory, Shenzhen Qianhai Shekou Free Trade Zone Hospital, Shenzhen, China; ^2^ Department of Pulmonary and Critical Care Medicine, Shenzhen Qianhai Shekou Free Trade Zone Hospital, Shenzhen, China

**Keywords:** *Acinetobacter baumannii*, carbapenem resistance, transcriptomic profiling, small RNA (sRNA), target gene

## Abstract

**Introduction:**

*Acinetobacter baumannii* (AB) is rising as a human pathogen of critical priority worldwide as it is the leading cause of opportunistic infections in healthcare settings and carbapenem-resistant AB is listed as a “super bacterium” or “priority pathogen for drug resistance” by the World Health Organization.

**Methods:**

Clinical isolates of *A. baumannii* were collected and tested for antimicrobial susceptibility. Among them, carbapenem-resistant and carbapenem-sensitive *A. baumannii* were subjected to prokaryotic transcriptome sequencing. The change of sRNA and mRNA expression was analyzed by bioinformatics and validated by quantitative reverse transcription-PCR.

**Results:**

A total of 687 clinical isolates were collected, of which 336 strains of *A. baumannii* were resistant to carbapenem. Five hundred and six differentially expressed genes and nineteen differentially expressed sRNA candidates were discovered through transcriptomic profile analysis between carbapenem-resistant isolates and carbapenem-sensitive isolates. Possible binding sites were predicted through software for sRNA21 and *adeK*, sRNA27 and *pgaC*, sRNA29 and *adeB*, sRNA36 and *katG*, indicating a possible targeting relationship. A negative correlation was shown between sRNA21 and *adeK* (r = -0.581, P = 0.007), sRNA27 and *pgaC* (r = -0.612, P = 0.004), sRNA29 and *adeB* (r = -0.516, P = 0.020).

**Discussion:**

This study preliminarily screened differentially expressed mRNA and sRNA in carbapenem-resistant *A. baumannii*, and explored possible targeting relationships, which will help further reveal the resistance mechanism and provide a theoretical basis for the development of drugs targeting sRNA for the prevention and treatment of carbapenem-resistant *A. baumannii* infection.

## Introduction

*Acinetobacter baumannii* (AB) is a gram-negative bacterial pathogen that is a common source of drug-resistant nosocomial infections. It can cause a wide range of hospital infections, especially in intensive care units (ICU), immunocompromised patients, and newborns. According to the 2018 China Antibacterial Surveillance Network (CHINET) study on clinical isolates of bacteria from 44 hospitals in major regions of China, 23573 strains of *Acinetobacter* were isolated clinically, with *A. baumannii* accounting for 92.5%, ranking first among non-fermenting bacteria (Hu et al., 2019). *A. baumannii* can cause meningitis, peritonitis, endocarditis, urinary system infections, as well as skin and soft tissue infections. The most common and highest mortality manifestations were ventilator-associated pneumonia (VAP) and bloodstream infections (BSI) (Antunes et al., 2014; Magill et al., 2014). Ventilator-associated pneumonia is one of the most common hospital infections among patients receiving mechanical ventilation. The occurrence of VAP prolongs the hospitalization time of ICU patients, and is accompanied by higher medical costs and a poorer prognosis. *A. baumannii* is considered the main pathogen causing high mortality in VAP patients in ICU (Čiginskienė et al., 2019). The infection of *A. baumannii* was significantly associated with an increase in mortality. The clinical manifestations of *A. baumannii* bloodstream infection can range from benign transient bacteremia to fulminant diseases and septic shock, with a total mortality rate of up to 46% (Jang et al., 2009).

With the widespread use of antibiotics, drug resistant strains of *A. baumannii* are on the rise. Carbapenem-resistant *A. baumannii* (CRAB) infections are extremely treatment resistant, often having no alternative antibiotics, creating a life threating situation for patients. In 2017, the World Health Organization (WHO) listed carbapenem-resistant *A. baumannii* as a “super bacterium” and included it in the first batch of “priority pathogens for drug resistance” (Tacconelli et al., 2018). Since Go et al. (1994) first reported the clinical isolation of CRAB strains in 1994, various clinical infectious diseases caused by CRAB strains have been reported all over the world. The resistance rate of *A. baumannii* to carbapenem antibiotics showed a rapid growth trend from 2005 to 2018, with resistance rates to meropenem and imipenem reaching 78.1% and 77.1%, respectively in 2018 (Hu et al., 2019). The resistance rate of *Acinetobacter* genus to meropenem in European and American countries is 79.9% from the SENTRY Antimicrobial Surveillance Program (Sader et al., 2016).

Bacterial small RNAs (sRNAs) are non-coding RNAs that regulate the expression of target genes, with a length of approximately 40–500 nucleotides. Small RNAs regulate many biological processes such as outer membrane protein biogenesis (Grabowicz et al., 2016), iron homeostasis (Chareyre et al., 2019), quorum sensing (Thomason et al., 2019), bacterial virulence (Sauder and Kendall, 2021), and antibiotic resistance (Noto et al., 2019) by binding to target mRNAs. The largest class of sRNAs are Hfq-dependent and modulate target mRNA translation or stability following direct binding through complimentary base pairing (Kavita et al., 2018). Recent studies have shown that abundant mRNA in bacteria is regulated by sRNA (Hör and Vogel, 2017; Desgranges et al., 2019; Djapgne and Oglesby, 2021). Small RNA regulation reduces energy loss for bacterial survival metabolism, provides more precise and faster gene regulation, and is beneficial for bacteria to adapt to new environmental pressures (Beisel and Storz, 2010). At present, sRNA-mediated gene regulation is considered a target for RNA drug development. Na et al. (2013) synthesized several sRNAs targeting different mRNA ribosomal binding sites (RBS) to regulate the expression of different genes in *Escherichia coli* strains. Small RNAs have also been widely used in bacterial metabolic engineering and synthetic biology. Candidate genes with clinical significance in infection and treatment, such as bacterial virulence and resistance genes, as well as mobile elements, have also become potential research targets for sRNAs. Cafiso et al. (2019) validated the differential expression of AbaRNA3 and MicrosRNA5 in polymyxin resistant *A. baumannii* using bioinformatics and qPCR methods. However, further research is needed on the biological function of this sRNA. According to reports, sRNAs in *Escherichia coli* affect the drug resistance by participating in antibiotic uptake (GcvB, RybB, MicF), drug efflux (DsrA, RydC, SdsR), biofilm formation (RprA, OmrA/B, McaS), lipopolysaccharide modification, and cell wall synthesis (MgrR, MicA) (Noto et al., 2019). The vast majority of mechanistic studies on sRNAs have been done in *E. coli* (Dersch et al., 2017). However, there have been limited studies executed to on the identification and characterization of *A. baumannii* sRNAs. This study will preliminarily screen sRNAs related to carbapenem resistance genes in *A. baumannii* and speculate on possible target genes and drug resistance mechanisms.

## Methods

### Bacterial strains, growth conditions and antimicrobial susceptibility

*Acinetobacter baumannii* strains were isolated from different patients at Shenzhen Qianhai Shekou Free Trade Zone Hospital. All strains were grown at 37°C with 5% CO2 and stored at −80°C in Luria-Bertani (LB) broth containing 10% glycerol. The isolates were identified using a VITEK2 automated instrument for ID/AST testing (bioMérieux, France). The susceptibility of *A. baumannii* strains to antimicrobial agents was determined using a microdilution method in accordance with the guidelines of the Clinical and Laboratory Standards Institute (Weinstein and Lewis, 2020).

### Total RNA extraction

The clinical isolates of *A. baumannii* were cultured in nutrition agar medium and the collected cells were subjected to centrifugation with a centrifugation speed of 2600 × g for 10 min at 4°C. The harvested pellets were washed twice with 5 mL of chilled sterile phosphate−buffered saline solution, and the pellets were treated with Trizol reagent (Ambion, Austin, TX, USA). The suspension was added to a screw-top tube containing sterile beads and lysed using a Bead Ruptor (Aoran, Shanghai, China). The Trizol solution was transferred to RNase-free tubes containing chloroform. The total RNA was further precipitated by isopropanol, 3-M sodium acetate, and glycogen for 10 min at room temperature. A second purification of the isolated total RNA was carried out with acidic phenol chloroform. Genomic DNA was removed by using RNase-free DNase I (Takara, Shiga, Japan) at 37°C for 3 h. RNA purity (OD260/OD280, OD260/OD230) was checked using the NanoPhotometer^®^ spectrophotometer (IMPLEN, CA, USA). The integrity of the purified RNA was assessed using the RNA Nano 6000 Assay Kit of the Bioanalyzer 2100 system (Agilent, Santa Clara, CA, USA) and the quality was monitored on 1% agarose gels.

### Library construction and sequencing

Firstly, the rRNA was depleted from 1 microgram of total RNA using Illumina MRZB12424 Ribo-Zero rRNA Removal Kit (Bacteria) (Illumina, San Diego, CA, USA). Then, the first-strand cDNA was synthesized using ProtoScript Reverse Transcriptase (New England BioLabs, Ipswich, MA, USA) at 25°C for 10 min; 42°C for 15 min; 70°C for 15 min. The second-strand cDNA was synthesized using NEBNext Second Strand Synthesis Reaction Buffer and dATP, dGTP, dCTP, dUTP mix (New England BioLabs, Ipswich, MA, USA) at 16°C for 1 h. Each preparation of cDNA was purified with Agencourt AMPure XP beads (Beckman Coulter, Brea, CA) and end repaired with NEBNext End Repair Reaction Buffer and Enzyme Mix (New England BioLabs, Ipswich, MA, USA) at 20°C for 30 min; 65°C for 30 min. Sequencing adapters were ligated using NEBNext Adaptor for Illumina (New England BioLabs, Ipswich, MA, USA) at 20°C for 15 min. The second-strand cDNA was then degraded using the USER enzyme mix (New England BioLabs, Ipswich, MA, USA) at 37°C for 15 min and the product was purified with Agencourt AMPure XP beads (Beckman Coulter, Brea, CA). Finally, the clustering of the index-coded samples was performed on a cBot Cluster Generation System using NEBNext Q5 Hot Start HiFi PCR Master Mix (New England Biolabs, Ipswich, MA, USA). After cluster generation, sequencing was performed using the Illumina Novaseq 6000 platform with pair-end 150 base reads.

### Clean reads filtering

Raw data were filtered by the following standards: 1) removing reads with 10% unidentified nucleotides (N); 2) removing reads with > 50% bases having phred quality scores of 20; 3) removing reads aligned to the barcode adapter using FASTP (version 0.18.0) (Chen S. et al., 2018). Quality trimmed reads were mapped to the reference genome using Bowtie2 (Langmead and Salzberg, 2012) (version 2.2.8) allowing no mismatches. Reads mapped to ribosome RNA were removed. Retainted reads were aligned with the reference genome using Bowtie2 (version 2.2.8) to identify known genes and calculated gene expression by RSEM (Li and Dewey, 2011).

### Analysis of replicate correlation and principle component

To evaluate reproducibility between samples, the correlation coefficient among replicas was calculated. Values closer to one indicates better reproducibility. Principle component analysis (PCA) was performed with the R package gmodels (http://www.r-project.org) to reveal the relationship between samples.

### Analysis of differentially expressed genes and sRNA

The gene expression level was further normalized by using the fragments per kilobase of transcript per million (FPKM) mapped reads method to eliminate the influence of different gene lengths and amounts of sequencing data on the calculation of gene expression. The edgeR package (http://www.r-project.org/) was used to identify differentially expressed genes (DEGs) across samples with fold changes 2 and a false discovery rate-adjusted *P* < 0.05. DEGs were then subjected to an enrichment analysis of GO function and KEGG pathways, and q values < 0.05 were used as a threshold.

All sRNA candidates were predicted in silico according to the method described previously (Mortazavi et al., 2008; Liu et al., 2016). The prediction of sRNA was conducted using Rockhopper (Robinson et al., 2010) (version 2.0.3) with the removal of sequences less than 50 bp. The annotation of novel transcripts was performed by aligning them to sequences in the NCBI NR database using BLASTX. Novel transcripts with NR annotations were considered novel potential protein-coding transcripts, and these could not be blasted against the NR databases that were considered to be sRNA candidates. RNAfold and IntaRNA were used to predict the secondary structure and target gene, respectively (Busch et al., 2008). The sRNA expression level was normalized by using TPM values (transcripts per million). The edgeR package (http://www.r-project.org/) was used to identify differentially expressed sRNAs across samples with fold changes >= 2 and a false discovery rate-adjusted *P* < 0.05.

### Quantitative reverse transcription-PCR

RNA extraction was performed as described previously and reverse-transcribed to cDNA using HiScript II Q RT SuperMix for qPCR (+gDNA wiper) (Vazyme, Nanjing, China) or miRNA First Strand cDNA Synthesis (Stem-loop Method) (Sangon, Shanghai, China). The qRT-PCR experiments were performed using SYBR Select Master Mix kit (Invitrogen, Carlsbad, USA) in accordance with the manufacturer’s instructions. The reaction was performed in a QuantStudio™ Real-Time PCR System (Applied Biosystems, Foster City, CA, USA) under the following conditions: 95°C for 10 min; 40 cycles of 95°C for 10 s, 58°C for 60 s. 16S rRNA gene was included as a reference for normalization of the gene expression data. The expression levels of sRNA and target genes in carbapenem-sensitive *A. baumannii* serve as controls. Results were analyzed using the comparative critical threshold method (2^-ΔΔCT^) (Livak and Schmittgen, 2001). All primers used in the qRT-PCR assay are shown in **Supporting information, Data S1**.

### Statistical analysis

All the experiments were biologically repeated at least thrice with three technical replicates per assay. A two-tailed unpaired Student’s *t* test was used to perform statistical analysis. *P* < 0.05 was considered statistically significant. Prism, version 8 (GraphPad Software Inc., San Diego, CA, USA) was used to conduct analyses.

## Results

### Antimicrobial resistance levels of clinical isolates of *Acinetobacter baumannii*

We have currently collected 687 clinical isolates of *A. baumannii* from January 2022 to March 2023. All the clinical strains have completed drug resistance testing (**Table 1**). The drug sensitivity results showed that 336 strains of carbapenem resistant *A. baumannii* were also resistant to piperacillin/tazobactam, ciprofloxacin, ceftazidime, and cefepime. Three hundred and fifty-one strains of carbapenem sensitive *A. baumannii* were partially sensitive to levofloxacin, tobramycin, cefoperazone/sulbactam, or amikacin, while 687 strains of *A. baumannii* were all sensitive to polymyxin.

**Table 1:** Drug resistance testing of all clinical isolates of *A. baumanni* collected from January 2022 to March 2023 that were carbapenem-resistant *A. baumannii* (CRAB) and carbapenem-sensitive *A. baumannii* (CSAB).

Antibiotics	n (687)		CRAB (336)		CSAB (351)	
	R (%)	S (%)	R (%)	S (%)	R (%)	S (%)
Piperacillin/Tazobactam	403 (58.66%)	284 (41.34%)	336 (100.00%)	0 (0.00%)	67 (19.09%)	284 (80.91%)
Ciprofloxacin	394 (57.35%)	293 (42.65%)	336 (100.00%)	0 (0.00%)	58 (16.52%)	293 (83.48%)
Ceftazidime	387 (56.33%)	300 (43.67%)	336 (100.00%)	0 (0.00%)	51 (14.53%)	300 (85.47%)
Cefepime	378 (55.02%)	309 (44.98%)	336 (100.00%)	0 (0.00%)	42 (11.97%)	309 (88.03%)
Meropenem	336 (48.91%)	351 (51.09%)	336 (100.00%)	0 (0.00%)	0 (0.00%)	351 (100.00%)
Imipenem	335 (48.76%)	352 (51.24%)	335 (99.70%)	1 (0.30%)	0 (0.00%)	351 (100.00%)
Levofloxacin	349 (50.80%)	338 (49.20%)	322 (95.83%)	14 (4.17%)	27 (7.69%)	324 (92.31%)
Tobramycin	339 (49.34%)	348 (50.66%)	318 (94.64%)	18 (5.36%)	21 (5.98%)	330 (94.02%)
Cefoperazone/Sulbactam	281 (40.90%)	406 (59.10%)	273 (81.25%)	63 (18.75%)	8 (2.28%)	343 (97.72%)
Amikacin	270 (39.30%)	417 (60.70%)	265 (78.87%)	71 (21.13%)	5 (1.42%)	346 (98.58%)
Minocycline	126 (18.34%)	561 (81.66%)	126 (37.50%)	210 (62.50%)	0 (0.00%)	351 (100.00%)
Tigecycline	5 (0.73%)	682 (99.27%)	5 (1.49%)	331 (98.51%)	0 (0.00%)	351 (100.00%)
Polymyxin	0 (0.00%)	687 (100.00%)	0 (0.00%)	336 (100.00%)	0 (0.00%)	351 (100.00%)

R, resistance; S, sensitiveness.

### Genome-wide differential gene expression in carbapenem-resistant and carbapenem-sensitive *Acinetobacter baumannii*

**Figure 1:**
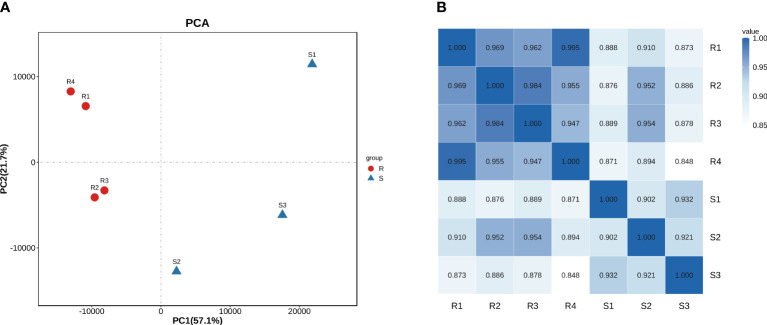
Principal component analysis **(A)** and heat map **(B)** on correlation strength of the clinical isolates of *A. baumannii* based on the transcriptomic profile.

**Figure 2:**
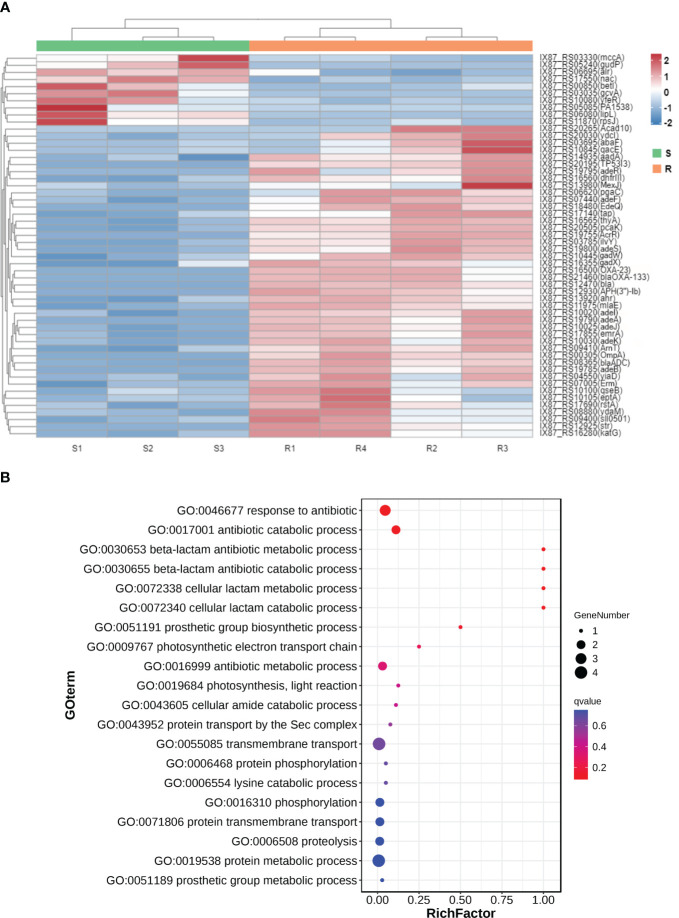
The mRNA expression heatmap **(A)** and functional annotation (GO) analysis **(B)** of 54 differentially expressed genes compared to the drug resistance database.

The transcriptomic profile between carbapenem-resistant isolates and carbapenem-sensitive isolates in clinic was analyzed by RNA-sequencing to investigate the differential gene expression profile in *A. baumannii* under drug resistance differences. Each group received four independent biological replicates. A sample of the carbapenem-sensitive group with a mapping rate of less than 80% to the reference genome was discarded. We reflected on the repeatability and correlation strength of the samples through principal component analysis (**Figure 1A**) and correlation heat map (**Figure 1B**), and the results showed significant differences between the two groups of specimens, with small intra-group differences and strong representativeness. In total, 506 differentially expressed genes were discovered through differential gene expression analysis, including 385 upregulated genes and 121 downregulated genes (see **Supporting information**, **Supplementary Figure S1**). We aligned these 506 differentially expressed gene sequences to the CARD resistance database (The comprehensive antimicrobial resistance database, https://card.mcmaster.ca/). A total of 54 differentially expressed genes were successfully compared to the drug resistance database (**Figure 2A**). We conducted functional annotation (GO) analysis on these 54 genes (**Figure 2B**), and the differentially expressed genes were mainly enriched in their functions in response to antibiotics, antibiotic catabolism beta-lactam metabolism process, etc.

### Genome-wide transcriptome identification of novel small RNAs in carbapenem-resistant *Acinetobacter baumannii*

Intergenic regions were considered as the possible source of novel transcripts. We used Rockhopper software to assemble the RNA-seq reads based on the reference genome and compare them with the annotated gene model to predict novel transcript regions. There were 92 new transcripts discovered, including 40 sRNA candidates (see **Supporting information, Data S2**) and 52 new potential coding transcripts (see **Supporting information, Data S3**). The FPKM of sRNA candidates for each sample is shown in **Supporting information, Data S2**, and the complete list of sRNAs includes their sequences, read counts, fold changes and adjusted *P*-values. The lengths of the 40 sRNA candidates range from 56 to 330 nucleotides. Nineteen differentially expressed sRNA candidates were identified in bacterial resistance to carbapenem (adjusted P-values (P-adj) < 0.05; │log2(fold change)│≥ 1). The different transcription levels of these 19 sRNAs in the carbapenem-sensitive and carbapenem-resistant groups suggest that they are likely to be involved in the posttranscriptional regulation of resistance to carbapenem antibiotic stress in *A. baumannii*.

Bacterial sRNA typically has multiple target mRNAs, and the same mRNA may be regulated by various non-coding sRNAs, resulting in a complex network of post-transcriptional regulation. The specific functions of sRNAs are difficult to define, and more extensive experiments are required. In the present study, we used a computer-based approach to predict the targets and regulatory networks of sRNA candidates. The target mRNA prediction of sRNA candidates was performed using targetRNA3 and IntaRNA, and the results are shown in are shown in **Supporting information, Data S4**. The secondary structure prediction results show that nineteen candidate sRNAs have high loop GC content, allowing these sRNAs to be more tightly and precisely linked to their target genes (see **Supporting information, Data S5**).

### *In silico* identification of mRNA targets and pathways regulated by sRNA

**Figure 3:**
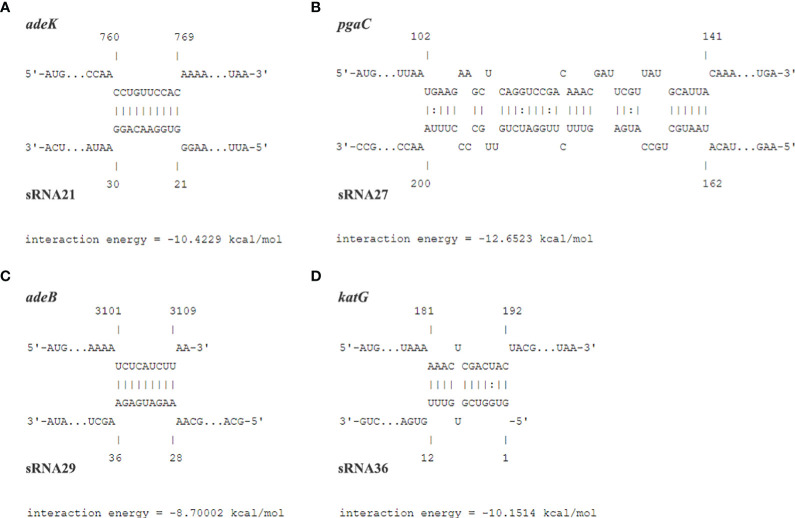
The predicted potential binding sites and the interaction energy between sRNA21 and *adeK* **(A)**, sRNA27 and *pgaC* **(B)**, sRNA29 and *adeB* **(C)**, sRNA36 and *katG* **(D)**.

**Figure 4:**
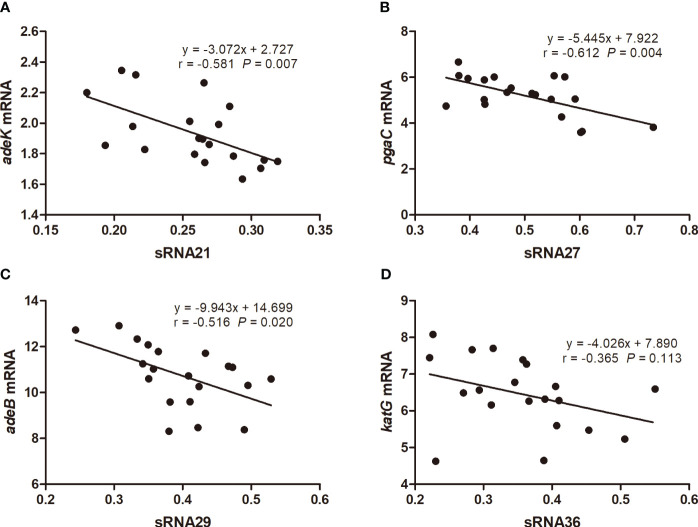
The correlation of relative expression levels between sRNA21 and *adeK* **(A)**, sRNA27 and *pgaC* **(B)**, sRNA29 and *adeB* **(C)**, sRNA36 and *katG* **(D)**. The numbers on the X or Y axes are the ratio of the relative expression level of sRNA or target gene mRNA in carbapenem-resistant *A. baumannii* to the relative expression level in carbapenem-sensitive *A. baumannii*.

We used prediction tools from the IntaRNA website and targetRNA3 website to predict the interactions and potential binding sites between 54 genes aligned to the CARD resistance database and 19 sRNAs. Both databases have predicted potential binding sites for sRNA21 and *adeK*, sRNA27 and *pgaC*, sRNA29 and *adeB*, sRNA36 and *katG*. The target genes were predicted using IntaRNA, which calculates the combined energy score of the interaction as the sum of the free energy of hybridization and the free energy required to make the interaction sites accessible (**Figure 3**). We used 20 clinical carbapenem resistant and 20 sensitive strains each to perform RNA extraction and qRT-PCR to detect the relative expression levels of sRNA21, sRNA27, sRNA29, sRNA36, *adeK*, *pgaC*, *adeB*, and *katG* (**Figure 4**). In **Figure 4**, there was showed a negative correlation between sRNA21 and *adeK* (r = -0.581, *P* = 0.007), sRNA27 and *pgaC* (r = -0.612, *P* = 0.004), sRNA29 and *adeB* (r = -0.516, *P* = 0.020). This means that the decrease in sRNA reduced the degree of inhibition of the target gene, leading to an increase in the expression level of the target gene. There was no correlation between the relative expression levels of sRNA36 and *katG* (r = -0.365, *P* = 0.113).

## Discussion

The high isolation rate and high resistance rate of *A. baumannii* seriously endanger human health and increase the burden of medical resources. Therefore, controlling the formation of drug-resistant strains and inhibiting the spread of bacterial resistance are urgent public health issues that need to be addressed in China and even globally. Chen Y. et al. (2018) collected 86 strains of *A. baumannii* isolated from the intensive care unit of the First Affiliated Hospital of Sun Yat Sen University, including 66 strains of MDRAB, with a resistance rate of up to 100% to imipenem. Over the past decade, sRNAs have been identified in a wide range of bacteria and found to play critical regulatory roles in bacterial life processes including bacterial resistance to antibiotics. Several sRNAs have been reported to regulate the expression of drug resistance genes to change drug resistance levels (Moon and Gottesman, 2009; Nishino et al., 2011; Acuña et al., 2016; Serra et al., 2016). The sRNA MgrR controls modification of the cell envelope and thereby mediates susceptibility of *E. coli* to the cationic antimicrobial peptide polymyxin B (Moon and Gottesman, 2009). The sRNA RybB targets the mRNA encoding the crucial biofilm regulator CsgD in *E. coli*, inhibits biofilm formation and affects biofilm-mediated resistance against antibiotics and host defenses (Serra et al., 2016). In this study, the transcriptomic profile between carbapenem-resistant isolates and carbapenem-sensitive isolates in the clinic was compared by RNA-sequencing to find out differential expression of several sRNAs and some mRNAs. 506 differentially expressed genes were discovered through differential gene expression analysis, of which 54 differentially expressed genes were successfully compared to the CARD resistance database. These drug resistance genes are mainly related to efflux pumps (*adeA, adeB, adeR, adeK*), hydrolases (*blaADC, tem*-1*, oxa*-23), biofilm synthesis (*pgaC, acrA, katG*), membrane protein synthesis (*ompA, yiaD, arpC*), and transcription factors (*ydcI, yfeR, betI*). Of these differentially expressed sRNAs, we predicted the target genes of a few novel sRNAs that may be related to the resistance mechanism of *A. baumannii*, and verified their correlation in clinical strains.

Recently, several sRNAs of *A. baumannii* have been identified through RNA-Seq or comparative genome analysis as potential regulators that might be involved in bacterial resistance to antibiotics. In this study, 19 sRNAs were identified as potential regulators by RNA-seq. The expressions of sRNAs were also validated by RT-PCR. Identification and characterization of new regulatory sRNAs can help understand how pathogens respond to various hosts’ microenvironments for their survival. *AdeK*, *pgaC*, *adeB*, and *katG* are target genes predicted by bioinformatics software for sRNA21, sRNA27, sRNA29, and sRNA36, respectively. Except for sRNA36 and *katG*, there is a negative correlation between the expression of other sRNAs and target genes. AdeB and AdeK are important proteins of the resistance/nodulation/division (RND) efflux pump superfamily, including AdeABC and AdeIJK. The function of AdeABC complex can be simply explained by the fact that AdeB captures substrates in the inner membrane of the phospholipids bilayer or the cytoplasm, then transports the substrates by AdeC (membrane channel protein). Therefore, these structural genes can promote drug discharge. Approximate 3.6-fold increases in *adeB* expression were observed in carbapenem-resistant *A. baumannii* compared to those in carbapenem-sensitive *A. baumannii*, which is similar to the results of previous study (Wong et al., 2009). In a separate study, *adeB* was inactivated by plasmid insertion and mutants for the *adeB* gene showed a 4- to 6-fold decrease in MICs for different antibiotics including carbapenem (meropenem) (Wong et al., 2009). The resistance−nodulation−cell−division−type multidrug efflux pump, AdeB, is not only associated with carbapenem resistance and has also been reported to be implicated in mediating the level of susceptibility towards other drugs, such as aminoglycoside, tetracyclines, chloramphenicol, erythromycin, trimethoprim, and ethidium bromide (Hou et al., 2012). The AdeABC efflux pump is regulated by AdeRS, a two-component system (TCS) consisting of a sensor kinase (AdeS) and a response regulator (AdeR) (Marchand et al., 2004). Mutations in *adeS* have been reported to affect phosphorylation activity (Lari et al., 2018), which is essential for activation of AdeR. AdeR binds to the intercistronic spacer (ICS) region and controls the expression of *adeB*. However, the loose binding of AdeR to ICS leads to overexpression of the AdeB efflux pump, resulting in drug resistance (Chang et al., 2016). AdeIJK is the fundamental RND system for species in the genus *Acinetobacter*. Expression of the AdeIJK efflux pump is regulated by AdeN which is a regulator protein belonging to the TetR family and the repressor of the *adeIJK* operon (Fernando et al., 2014). AdeIJK can export a broad range of antibiotics and provides crucial functions within the cell, for example lipid modulation of the cell membrane, and therefore it is likely that all *Acinetobacter* require AdeIJK for survival and homeostasis (Darby et al., 2023). In contrast, additional RND systems, such as AdeABC and AdeFGH, were only found in a subset of *Acinetobacter* that are associated with infection (Darby et al., 2023). AdeABC and AdeIJK efflux pumps have non-overlapping substrate specificities. Their inactivation leads to specific nonoverlapping changes in gene expression (Leus et al., 2020). Inactivation of AdeIJK elicits broad changes in the abundance of mRNAs and this response is modified in the absence of AdeB. In contrast, inactivation of AdeB leads to a focused cellular response, which is not sensitive to the activity of AdeIJK (Leus et al., 2020). A variety of efflux pump systems, including AdeABC, AdeIJK and AbeM, play important roles in the resistance to imipenem in *A. baumannii* (Hou et al., 2012). The PgaABCD operon encodes the proteins that produce Poly-β (1–6)-N-acetylglucosamine (PNAG), which is a surface polysaccharide and has been well portrayed as a major component of biofilms in *A. baumannii* (Maira-Litrán et al., 2002; Choi et al., 2009). As well, PNAG protects *A. baumannii* against innate host defenses (Choi et al., 2009). As an encoding gene involved in the synthesis of PNAG, *pgaC* gene expression could be down-regulated to improve the susceptibility of *A. baumannii* strains towards conventional antibiotics by pyrogallol (Abirami et al., 2023), phytol (Ramanathan et al., 2018), α-mangostin (Sivaranjani et al., 2018), myrtenol (Selvaraj et al., 2020).

There is some limitation for screening sRNA and mRNA related to carbapenem resistance. We could not collect clinical strains that were only resistant to carbapenems, even if we could collect wild strains that were sensitive to clinical antibiotics as control. Therefore, the experimental group and control group were only selected based on the definition of carbapenem-resistant strains rather than clinical strains with the same drug sensitivity except for differences in carbapenem resistance. The screening of sRNA and mRNA related to carbapenem resistance might have certain limitations. However, with more research and samples added, we believe that more reliable sRNA related strongly to carbapenem resistance will be screened out. We will also artificially induce carbapenem-sensitive strains into carbapenem-resistant strains, and refer to the results of this study to further screen sRNA and determine its biological function.

In summary, transcriptomic profiling of carbapenem-resistant *A. baumannii* revealed significant alterations in sRNA and mRNA expression. The differentially expressed sRNAs were predicted to be primarily involved in the drug resistance of *A. baumannii*. Our study represented that sRNA21, sRNA27, and sRNA29 could modulate the expression of the predicted target genes (*adeK*, *pgaC*, and *adeB*). Further analysis of sRNA functions will help us better understand the molecular mechanisms of drug resistance in *A. baumannii*. Further experiments are needed to disclose the precise mechanisms of how these sRNAs are implicated in bacterial resistance to antimicrobial drugs.

## Data availability statement

The original contributions presented in the study are included in the article/**Supplementary Material**. Further inquiries can be directed to the corresponding authors.

## Ethics statement

This work was approved by the Ethics Committee of Shenzhen Qianhai Shekou Free Trade Zone Hospital, and informed consent was obtained from all subjects.

## Author contributions

YW: Writing – original draft, Writing – review & editing, Conceptualization, Data curation, Formal Analysis, Funding acquisition, Investigation, Methodology, Project administration, Software. XX: Data curation, Formal Analysis, Investigation, Methodology, Software, Writing – review & editing. JZ: Data curation, Formal Analysis, Investigation, Methodology, Software, Writing – review & editing. QL: Data curation, Investigation, Methodology, Resources, Writing – review & editing. YR: Data curation, Investigation, Methodology, Resources, Writing – review & editing. YZ: Data curation, Investigation, Methodology, Writing – review & editing. MZ: Data curation, Investigation, Methodology, Writing – review & editing. JM: Resources, Supervision, Writing – review & editing. SH: Conceptualization, Formal Analysis, Methodology, Project administration, Resources, Supervision, Validation, Writing – review & editing.

## References

[B1] Abirami G. Alexpandi R. Sudhin S. Durgadevi R. Roshni P. S. Kumar P.. (2023). Pyrogallol downregulates the expression of virulence-associated proteins in *Acinetobacter baumannii* and showing anti-infection activity by improving non-specific immune response in zebrafish model. Int. J. Biol. Macromol. 226, 853–869. doi: 10.1016/j.ijbiomac.2022.12.045 36526063

[B2] Acuña L. G. Barros M. J. Peñaloza D. Rodas P. I. Paredes-Sabja D. Fuentes J. A.. (2016). A feed-forward loop between SroC and MgrR small RNAs modulates the expression of eptB and the susceptibility to polymyxin B in *Salmonella Typhimurium* . Microbiology 162, 1996–2004. doi: 10.1099/mic.0.000365 27571709

[B3] Antunes L. C. S. Visca P. Towner K. J. (2014). *Acinetobacter baumannii*: evolution of a global pathogen. Pathog. Dis. 71, 292–301. doi: 10.1111/2049-632X.12125 24376225

[B4] Beisel C. L. Storz G. (2010). Base pairing small RNAs and their roles in global regulatory networks. FEMS Microbiol. Rev. 34, 866–882. doi: 10.1111/j.1574-6976.2010.00241.x 20662934 PMC2920360

[B5] Busch A. Richter A. S. Backofen R. (2008). IntaRNA: efficient prediction of bacterial sRNA targets incorporating target site accessibility and seed regions. Bioinformatics 24, 2849–2856. doi: 10.1093/bioinformatics/btn544 18940824 PMC2639303

[B6] Cafiso V. Stracquadanio S. Lo Verde F. Dovere V. Zega A. Pigola G.. (2019). COL^R^ *Acinetobacter baumannii* sRNA signatures: Computational comparative identification and biological targets. Front. Microbiol. 10, 3075. doi: 10.3389/fmicb.2019.03075 32010115 PMC6978653

[B7] Chang T. Y. Huang B. J. Sun J. R. Perng C. L. Chan M. C. Yu C. P.. (2016). AdeR protein regulates adeABC expression by binding to a direct-repeat motif in the intercistronic spacer. Microbiol. Res. 183, 60–67. doi: 10.1016/j.micres.2015.11.010 26805619

[B8] Chareyre S. Barras F. Mandin P. (2019). A small RNA controls bacterial sensitivity to gentamicin during iron starvation. PloS Genet. 15, e1008078. doi: 10.1371/journal.pgen.1008078 31009454 PMC6497325

[B9] Chen S. Zhou Y. Chen Y. Gu J. (2018). fastp: an ultra-fast all-in-one FASTQ preprocessor. Bioinformatics 34, i884–i890. doi: 10.1093/bioinformatics/bty560 30423086 PMC6129281

[B10] Chen Y. Ai L. Guo P. Huang H. Wu Z. Liang X.. (2018). Molecular characterization of multidrug resistant strains of *Acinetobacter baumannii* isolated from pediatric intensive care unit in a Chinese tertiary hospital. BMC Infect. Dis. 18, 614. doi: 10.1186/s12879-018-3511-0 30509192 PMC6278058

[B11] Choi A. H. Slamti L. Avci F. Y. Pier G. B. Maira-Litrán T. (2009). The pgaABCD locus of *Acinetobacter baumannii* encodes the production of poly-beta-1-6-N-acetylglucosamine, which is critical for biofilm formation. J. Bacteriol. 191, 5953–5963. doi: 10.1128/JB.00647-09 19633088 PMC2747904

[B12] Čiginskienė A. Dambrauskienė A. Rello J. Adukauskienė D. (2019). Ventilator-associated pneumonia due to drug-resistant *Acinetobacter baumannii*: Risk factors and mortality relation with resistance profiles, and independent predictors of in-hospital mortality. Medicina 55, 49. doi: 10.3390/medicina55020049 30781896 PMC6410055

[B13] Darby E. M. Bavro V. N. Dunn S. Mcnally A. Blair J. M. A. (2023). RND pumps across the genus *Acinetobacter*: AdeIJK is the universal efflux pump. Microb. Genom. 9, mgen000964. doi: 10.1099/mgen.0.000964 36995182 PMC10132057

[B14] Dersch P. Khan M. A. Muhlen S. Gorke B. (2017). Roles of regulatory RNAs for antibiotic resistance in bacteria and their potential value as novel drug targets. Front. Microbiol. 8, 803. doi: 10.3389/fmicb.2017.00803 28529506 PMC5418344

[B15] Desgranges E. Marzi S. Moreau K. Romby P. Caldelari I. (2019). Noncoding RNA. Microbiol. Spectr. 7, 2. doi: 10.1128/microbiolspec.GPP3-0038-2018 PMC11590673 31004423

[B16] Djapgne L. Oglesby A. G. (2021). Impacts of small RNAs and their chaperones on bacterial pathogenicity. Front. Cell. Infect. Microbiol. 11, 604511. doi: 10.3389/fcimb.2021.604511 34322396 PMC8311930

[B17] Fernando D. M. Xu W. Loewen P. C. Zhanel G. G. Kumar A. (2014). Triclosan can select for an AdeIJK-overexpressing mutant of *Acinetobacter baumannii* ATCC 17978 that displays reduced susceptibility to multiple antibiotics. Antimicrob. Agents Chemother. 58, 6424–6431. doi: 10.1128/AAC.03074-14 25136007 PMC4249441

[B18] Go E. S. Urban C. Burns J. Kreiswirth B. Eisner W. Mariano N.. (1994). Clinical and molecular epidemiology of *Acinetobacter* infections sensitive only to polymyxin B and sulbactam. Lancet 344, 1329–1332. doi: 10.1016/S0140-6736(94)90694-7 7968028

[B19] Grabowicz M. Koren D. Silhavy T. J. (2016). The CpxQ sRNA negatively regulates skp to prevent mistargeting of β-barrel outer membrane proteins into the cytoplasmic membrane. mBio 7, e00312–e00316. doi: 10.1128/mBio.00312-16 27048800 PMC4817254

[B20] Hör J. Vogel J. (2017). Global snapshots of bacterial RNA networks. EMBO J. 36, 245–247. doi: 10.15252/embj.201696072 28031253 PMC5286387

[B21] Hou P. F. Chen X. Y. Yan G. F. Wang Y. P. Ying C. M. (2012). Study of the correlation of imipenem resistance with efflux pumps AdeABC, AdeIJK, AdeDE and AbeM in clinical isolates of *Acinetobacter baumannii* . Chemotherapy 58, 152–158. doi: 10.1159/000335599 22614896

[B22] Hu F. Guo Y. Yang Y. Zheng Y. Wu S. Jiang X.. (2019). Resistance reported from China antimicrobial surveillance network (CHINET) in 2018. Eur. J. Clin. Microbiol. Infect. Dis. 38, 2275–2281. doi: 10.1007/s10096-019-03673-1 31478103

[B23] Jang T. N. Lee S. H. Huang C. H. Lee C. L. Chen W. Y. (2009). Risk factors and impact of nosocomial *Acinetobacter baumannii* bloodstream infections in the adult intensive care unit: a case-control study. J. Hosp. Infect. 73, 143–150. doi: 10.1016/j.jhin.2009.06.007 19716203

[B24] Kavita K. De Mets F. Gottesman S. (2018). New aspects of RNA-based regulation by Hfq and its partner sRNAs. Curr. Opin. Microbiol. 42, 53–61. doi: 10.1016/j.mib.2017.10.014 29125938 PMC10367044

[B25] Langmead B. Salzberg S. L. (2012). Fast gapped-read alignment with Bowtie 2. Nat. Methods 9, 357–359. doi: 10.1038/nmeth.1923 22388286 PMC3322381

[B26] Lari A. R. Ardebili A. Hashemi A. (2018). AdeR-AdeS mutations & overexpression of the AdeABC efflux system in ciprofloxacin-resistant *Acinetobacter baumannii* clinical isolates. Indian J. Med. Res. 147, 413–421. doi: 10.4103/ijmr.IJMR_644_16 29998878 PMC6057251

[B27] Leus I. V. Adamiak J. Trinh A. N. Smith R. D. Smith L. Richardson S.. (2020). Inactivation of AdeABC and AdeIJK efflux pumps elicits specific nonoverlapping transcriptional and phenotypic responses in *Acinetobacter baumannii* . Mol. Microbiol. 114, 1049–1065. doi: 10.1111/mmi.14594 32858760

[B28] Li B. Dewey C. N. (2011). RSEM: accurate transcript quantification from RNA-Seq data with or without a reference genome. BMC Bioinf. 12, 323. doi: 10.1186/1471-2105-12-323 PMC3163565 21816040

[B29] Liu M. Zhu Z. T. Tao X. Y. Wang F. Q. Wei D. Z. (2016). RNA-Seq analysis uncovers non-coding small RNA system of *Mycobacterium neoaurum* in the metabolism of sterols to accumulate steroid intermediates. Microb. Cell Fact. 15, 64. doi: 10.1186/s12934-016-0462-2 27112590 PMC4845491

[B30] Livak K. J. Schmittgen T. D. (2001). Analysis of relative gene expression data using real-time quantitative PCR and the 2(-Delta Delta C(T)) Method. Methods 25, 402–408. doi: 10.1006/meth.2001.1262 11846609

[B31] Magill S. S. Edwards J. R. Bamberg W. Beldavs Z. G. Dumyati G. Kainer M. A.. (2014). Multistate point-prevalence survey of health care-associated infections. N. Engl. J. Med. 370, 1198–1208. doi: 10.1056/NEJMoa1306801 24670166 PMC4648343

[B32] Maira-Litrán T. Kropec A. Abeygunawardana C. Joyce J. Mark G. Goldmann D. A.. (2002). Immunochemical properties of the staphylococcal poly-N-acetylglucosamine surface polysaccharide. Infect. Immun. 70, 4433–4440. doi: 10.1128/IAI.70.8.4433-4440.2002 12117954 PMC128161

[B33] Marchand I. Damier-Piolle L. Courvalin P. Lambert T. (2004). Expression of the RND-type efflux pump AdeABC in *Acinetobacter baumannii* is regulated by the AdeRS two-component system. Antimicrob. Agents Chemother. 48, 3298–3304. doi: 10.1128/AAC.48.9.3298-3304.2004 15328088 PMC514774

[B34] Moon K. Gottesman S. (2009). A PhoQ/P-regulated small RNA regulates sensitivity of *Escherichia coli* to antimicrobial peptides. Mol. Microbiol. 74, 1314–1330. doi: 10.1111/j.1365-2958.2009.06944.x 19889087 PMC2841474

[B35] Mortazavi A. Williams B. A. Mccue K. Schaeffer L. Wold B. (2008). Mapping and quantifying mammalian transcriptomes by RNA-Seq. Nat. Methods 5, 621–628. doi: 10.1038/nmeth.1226 18516045 PMC13303166

[B36] Na D. Yoo S. M. Chung H. Park H. Park J. H. Lee S. Y. (2013). Metabolic engineering of *Escherichia coli* using synthetic small regulatory RNAs. Nat. Biotechnol. 31, 170–174. doi: 10.1038/nbt.2461 23334451

[B37] Nishino K. Yamasaki S. Hayashi-Nishino M. Yamaguchi A. (2011). Effect of overexpression of small non-coding DsrA RNA on multidrug efflux in *Escherichia coli* . J. Antimicrob. Chemother. 66, 291–296. doi: 10.1093/jac/dkq420 21088020

[B38] Noto G. P. D. Molina M. C. Quiroga C. (2019). Insights into non-coding RNAs as novel antimicrobial drugs. Front. Genet. 10, 57. doi: 10.3389/fgene.2019.00057 30853970 PMC6395445

[B39] Ramanathan S. Arunachalam K. Chandran S. Selvaraj R. Shunmugiah K. P. Arumugam V. R. (2018). Biofilm inhibitory efficiency of phytol in combination with cefotaxime against nosocomial pathogen *Acinetobacter baumannii* . J. Appl. Microbiol. 125, 56–71. doi: 10.1111/jam.2018.125.issue-1 29473983

[B40] Robinson M. D. Mccarthy D. J. Smyth G. K. (2010). edgeR: a Bioconductor package for differential expression analysis of digital gene expression data. Bioinformatics 26, 139–140. doi: 10.1093/bioinformatics/btp616 19910308 PMC2796818

[B41] Sader H. S. Castanheira M. Farrell D. J. Flamm R. K. Mendes R. E. Jones R. N. (2016). Tigecycline antimicrobial activity tested against clinical bacteria from Latin American medical centres: results from SENTRY Antimicrobial Surveillance Program, (2011-2014). Int. J. Antimicrob. Agents 48, 144–150. doi: 10.1016/j.ijantimicag.2016.04.021 27291285

[B42] Sauder A. B. Kendall M. M. (2021). A pathogen-specific sRNA influences enterohemorrhagic *Escherichia coli* fitness and virulence in part by direct interaction with the transcript encoding the ethanolamine utilization regulatory factor EutR. Nucleic Acids Res. 49, 10988–11004. doi: 10.1093/nar/gkab863 34591974 PMC8565329

[B43] Selvaraj A. Valliammai A. Sivasankar C. Suba M. Sakthivel G. Pandian S. K. (2020). Antibiofilm and antivirulence efficacy of myrtenol enhances the antibiotic susceptibility of *Acinetobacter baumannii* . Sci. Rep. 10, 21975. doi: 10.1038/s41598-020-79128-x 33319862 PMC7738676

[B44] Serra D. O. Mika F. Richter A. M. Hengge R. (2016). The green tea polyphenol EGCG inhibits E. coli biofilm formation by impairing amyloid curli fibre assembly and downregulating the biofilm regulator CsgD via the σ(E) -dependent sRNA RybB. Mol. Microbiol. 101, 136–151. doi: 10.1111/mmi.13379 26992034

[B45] Sivaranjani M. Srinivasan R. Aravindraja C. Karutha Pandian S. Veera Ravi A. (2018). Inhibitory effect of α-mangostin on *Acinetobacter baumannii* biofilms – an in *vitro* study. Biofouling 34, 579–593. doi: 10.1080/08927014.2018.1473387 29869541

[B46] Tacconelli E. Carrara E. Savoldi A. Harbarth S. Mendelson M. Monnet D. L.. (2018). Discovery, research, and development of new antibiotics: the WHO priority list of antibiotic-resistant bacteria and tuberculosis. Lancet Infect. Dis. 18, 318–327. doi: 10.1016/S1473-3099(17)30753-3 29276051

[B47] Thomason M. K. Voichek M. Dar D. Addis V. Fitzgerald D. Gottesman S.. (2019). A rhlI 5’ UTR-derived sRNA regulates RhlR-dependent quorum sensing in *Pseudomonas aeruginosa* . mBio 10, e02253–e02219. doi: 10.1128/mBio.02253-19 31594819 PMC6786874

[B48] Weinstein M. P. Lewis J. S. 2nd (2020). The Clinical and Laboratory Standards Institute Subcommittee on Antimicrobial Susceptibility Testing: background, organization, functions, and processes. J. Clin. Microbiol. 58, e01864–e01819. doi: 10.1128/JCM.01864-19 31915289 PMC7041576

[B49] Wong E. W. Yusof M. Y. Mansor M. B. Anbazhagan D. Ong S. Y. Sekaran S. D. (2009). Disruption of adeB gene has a greater effect on resistance to meropenems than adeA gene in *Acinetobacter* spp. isolated from University Malaya Medical Centre. Singapore Med. J. 50, 822–826. 19710984

