# Supplementary figures and images for: Genome-wide sRNA and mRNA transcriptomic profiling insights into carbapenem-resistant Acinetobacter baumannii
Source: Front Cell Infect Microbiol. 2024 Jul 30;14:1419989. doi: 10.3389/fcimb.2024.1419989 (PMC11362675; doi:10.3389/fcimb.2024.1419989)

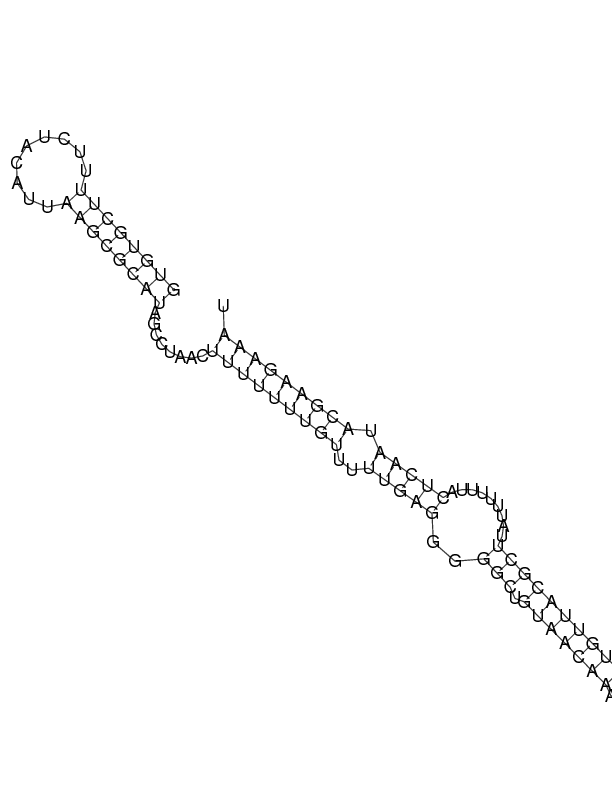

Supplement: Supplementary file 1 [file DataSheet1.zip › Data S5/sRNA14.png]

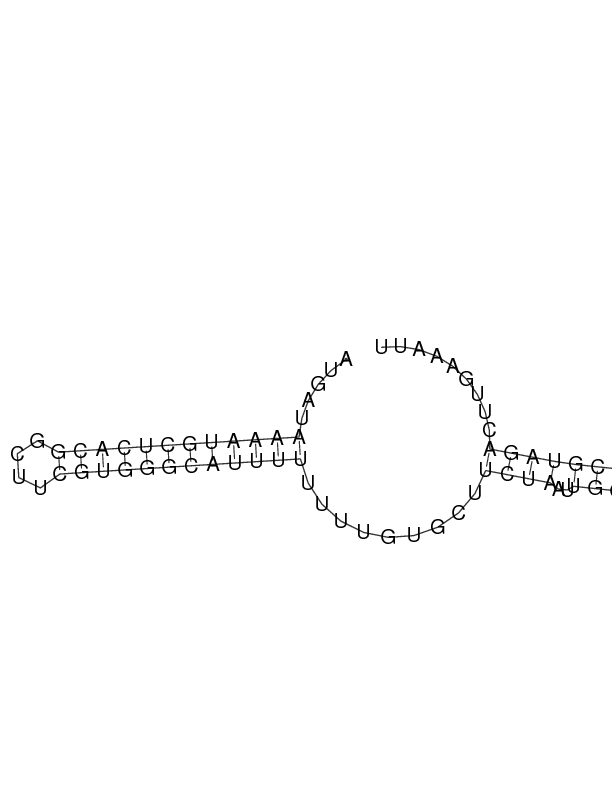

Supplement: Supplementary file 1 [file DataSheet1.zip › Data S5/sRNA15.png]

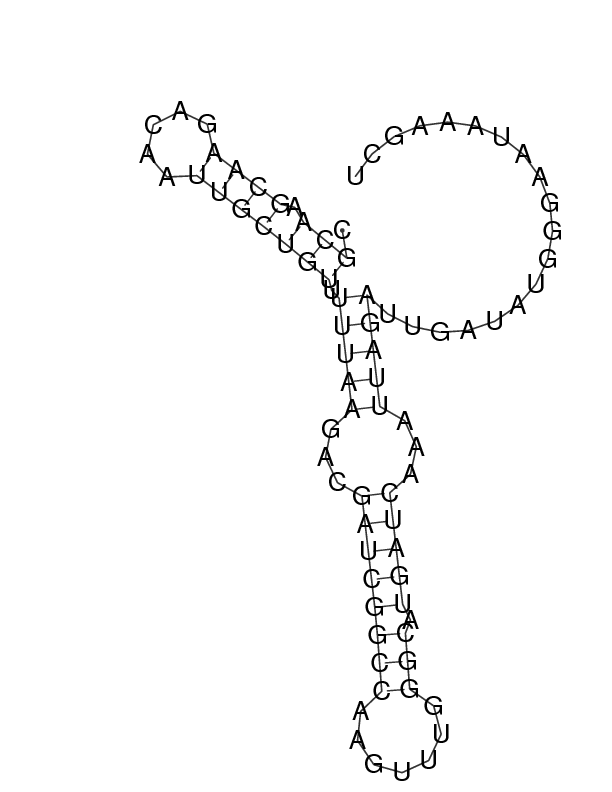

Supplement: Supplementary file 1 [file DataSheet1.zip › Data S5/sRNA19.png]

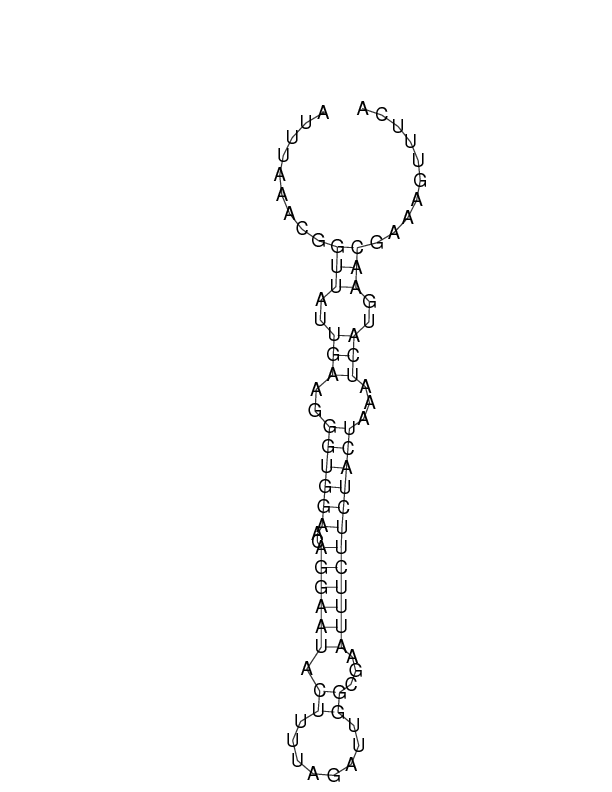

Supplement: Supplementary file 1 [file DataSheet1.zip › Data S5/sRNA21.png]

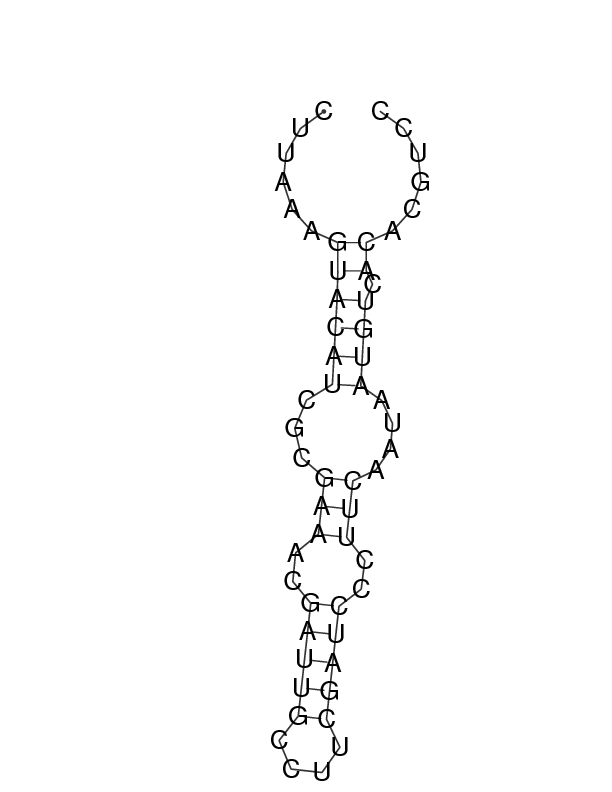

Supplement: Supplementary file 1 [file DataSheet1.zip › Data S5/sRNA22.png]

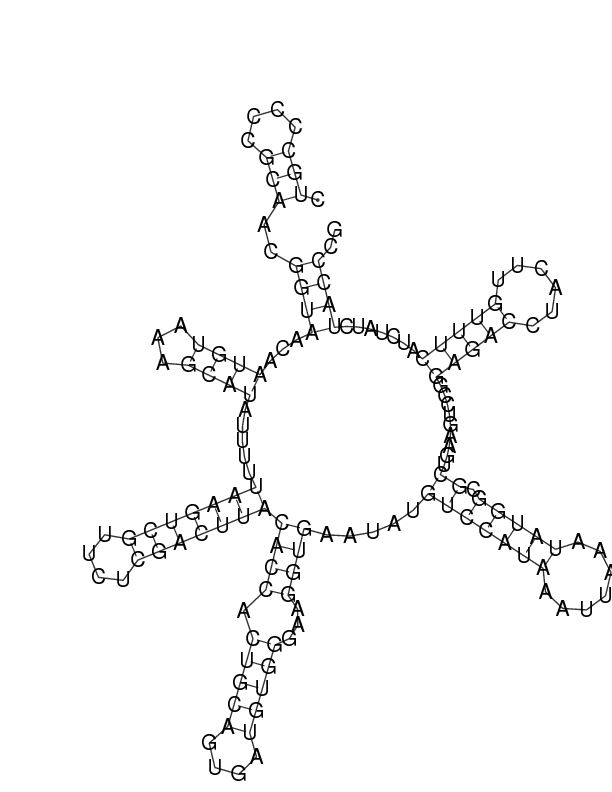

Supplement: Supplementary file 1 [file DataSheet1.zip › Data S5/sRNA23.png]

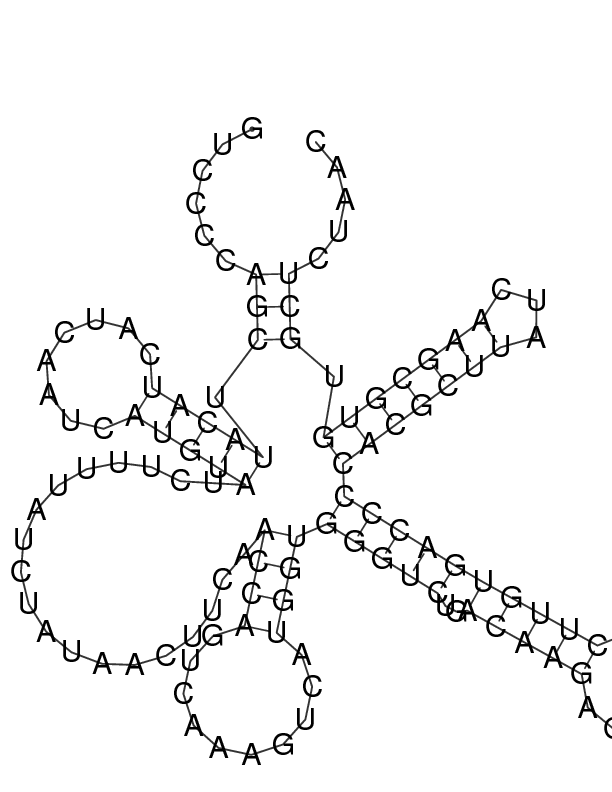

Supplement: Supplementary file 1 [file DataSheet1.zip › Data S5/sRNA25.png]

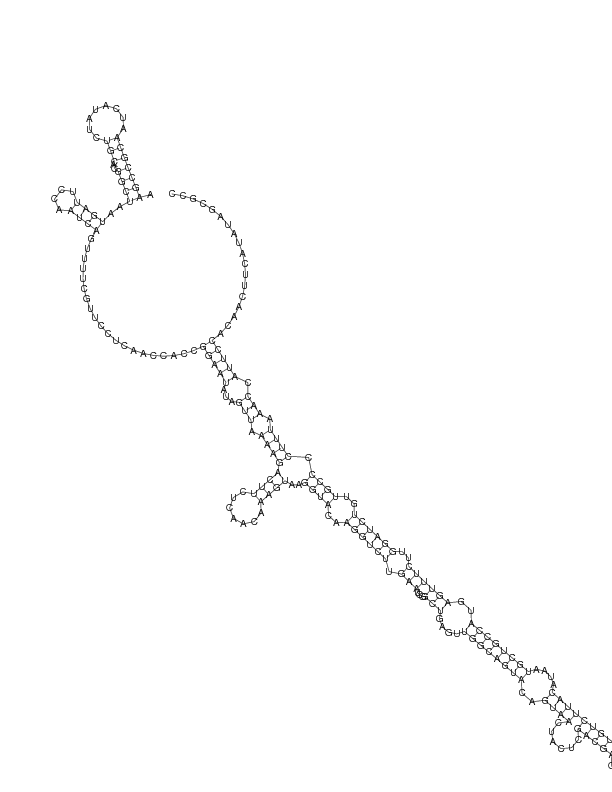

Supplement: Supplementary file 1 [file DataSheet1.zip › Data S5/sRNA27.png]

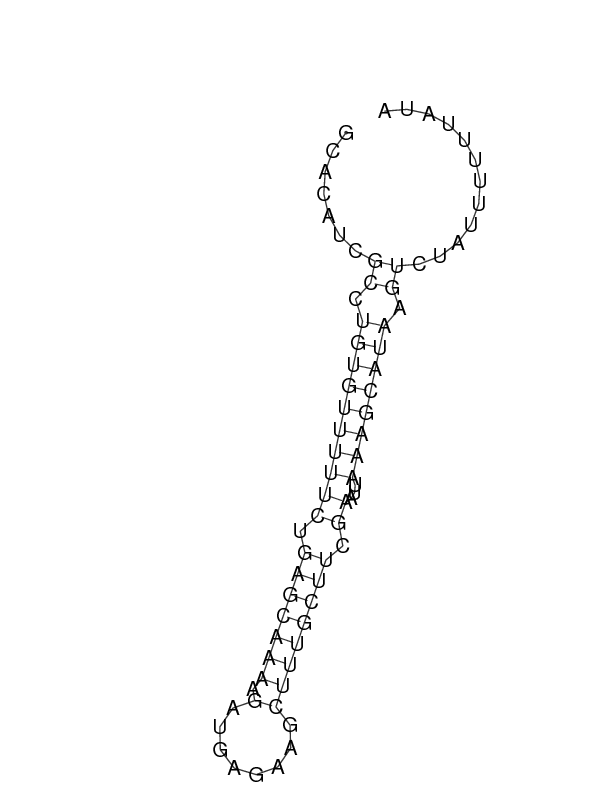

Supplement: Supplementary file 1 [file DataSheet1.zip › Data S5/sRNA29.png]

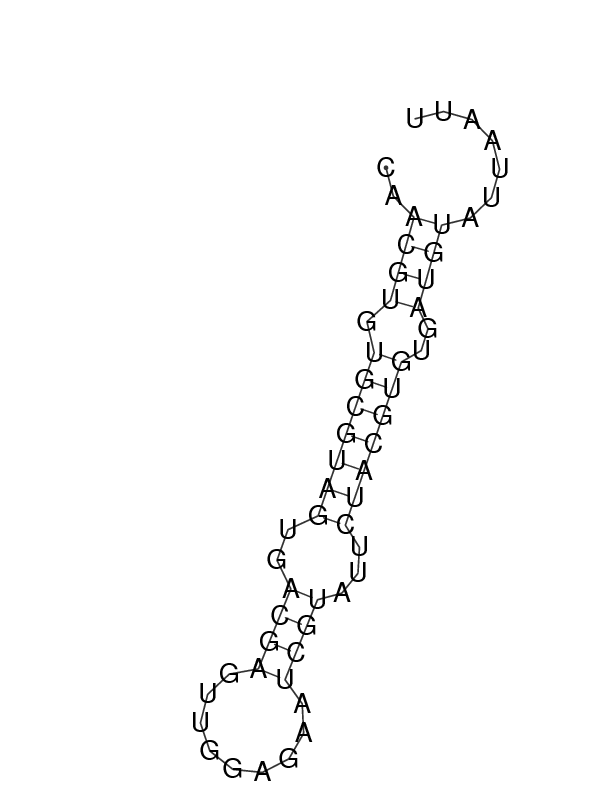

Supplement: Supplementary file 1 [file DataSheet1.zip › Data S5/sRNA3.png]

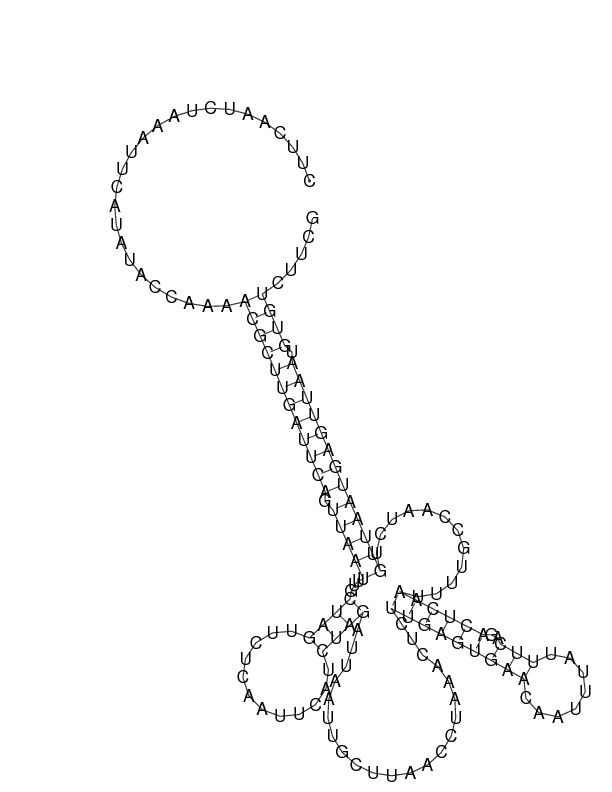

Supplement: Supplementary file 1 [file DataSheet1.zip › Data S5/sRNA32.png]

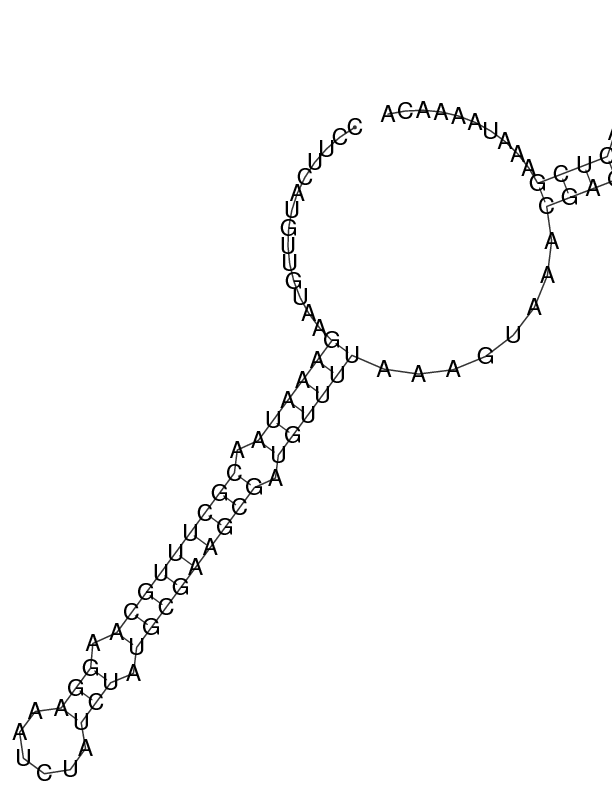

Supplement: Supplementary file 1 [file DataSheet1.zip › Data S5/sRNA33.png]

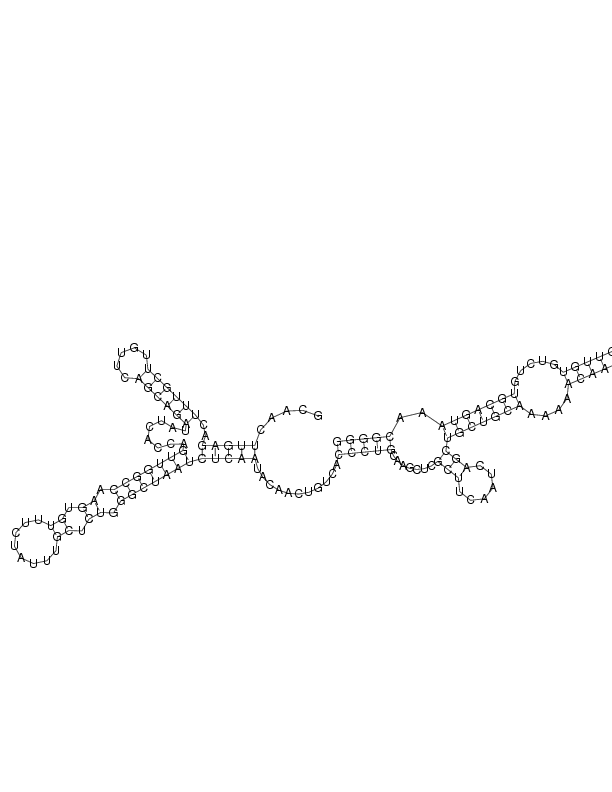

Supplement: Supplementary file 1 [file DataSheet1.zip › Data S5/sRNA34.png]

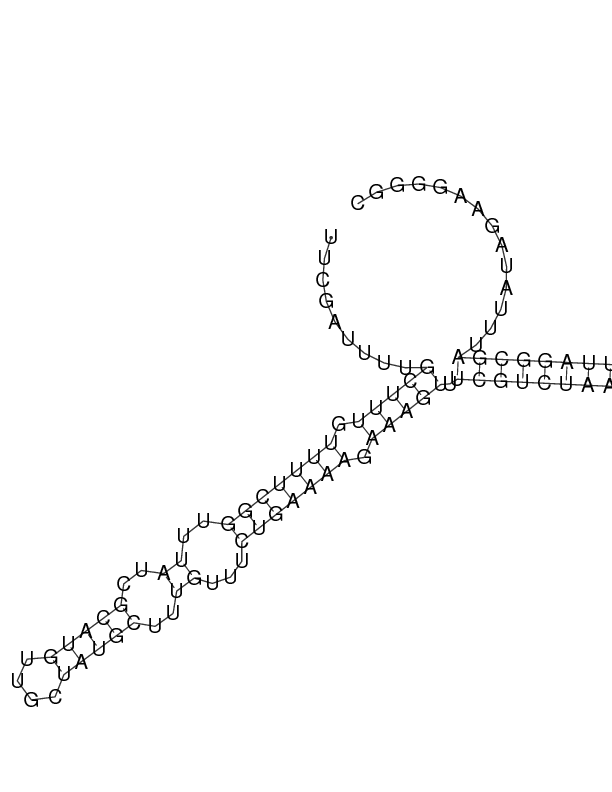

Supplement: Supplementary file 1 [file DataSheet1.zip › Data S5/sRNA35.png]

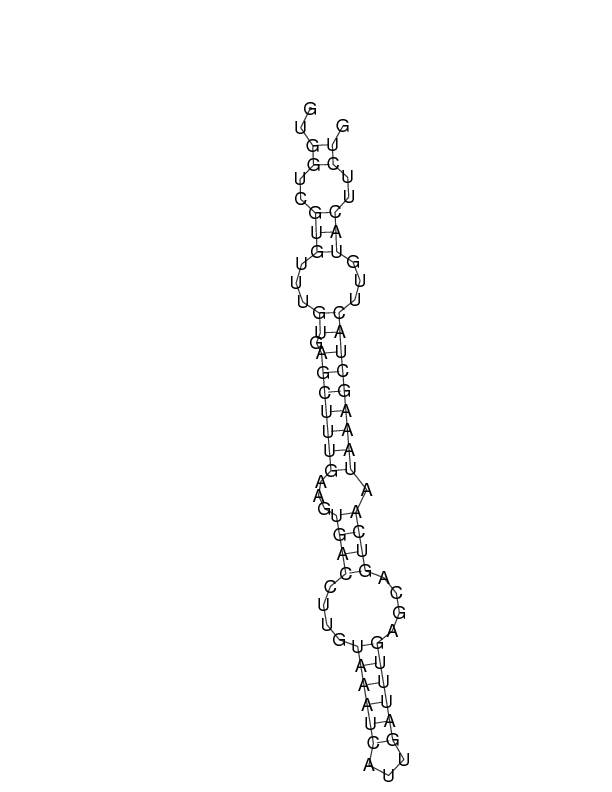

Supplement: Supplementary file 1 [file DataSheet1.zip › Data S5/sRNA36.png]

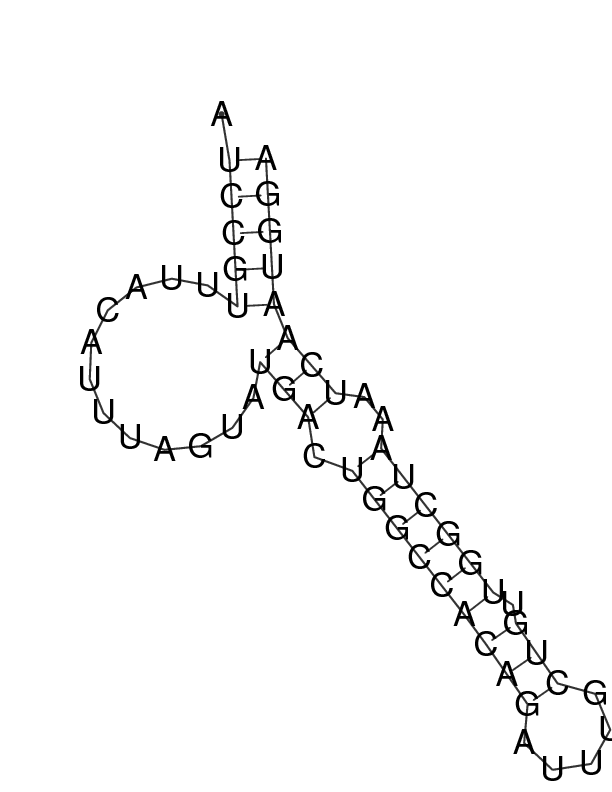

Supplement: Supplementary file 1 [file DataSheet1.zip › Data S5/sRNA38.png]

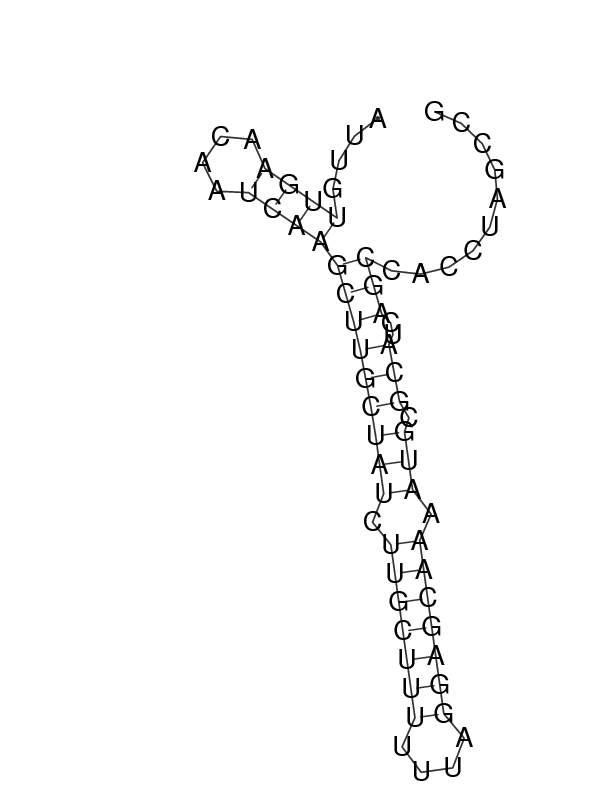

Supplement: Supplementary file 1 [file DataSheet1.zip › Data S5/sRNA39.png]

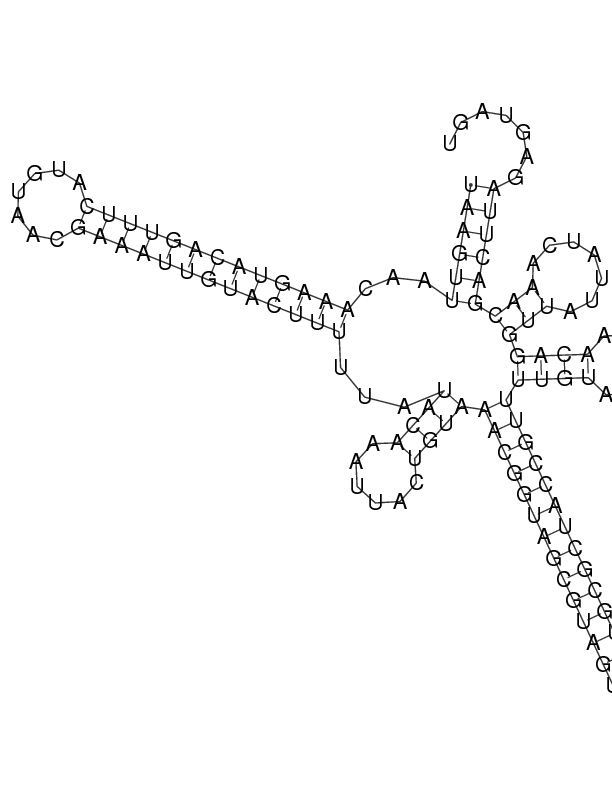

Supplement: Supplementary file 1 [file DataSheet1.zip › Data S5/sRNA4.png]

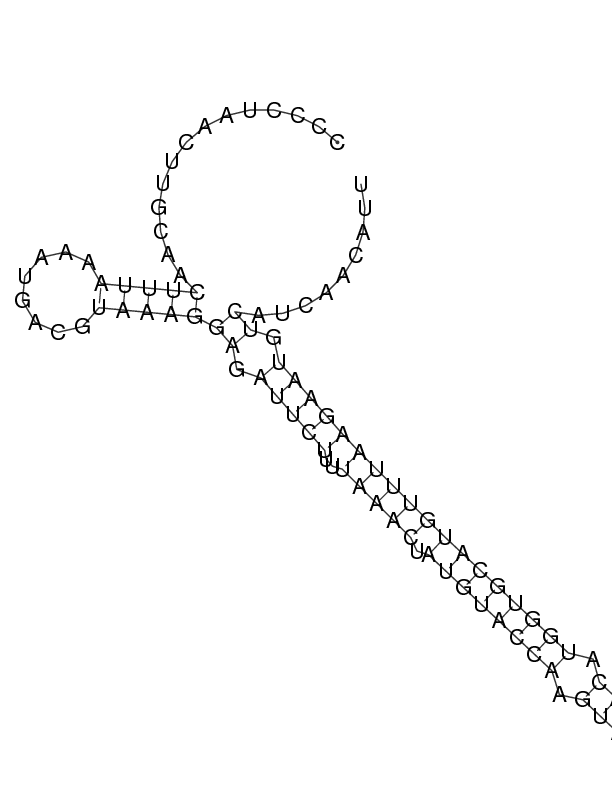

Supplement: Supplementary file 1 [file DataSheet1.zip › Data S5/sRNA40.png]

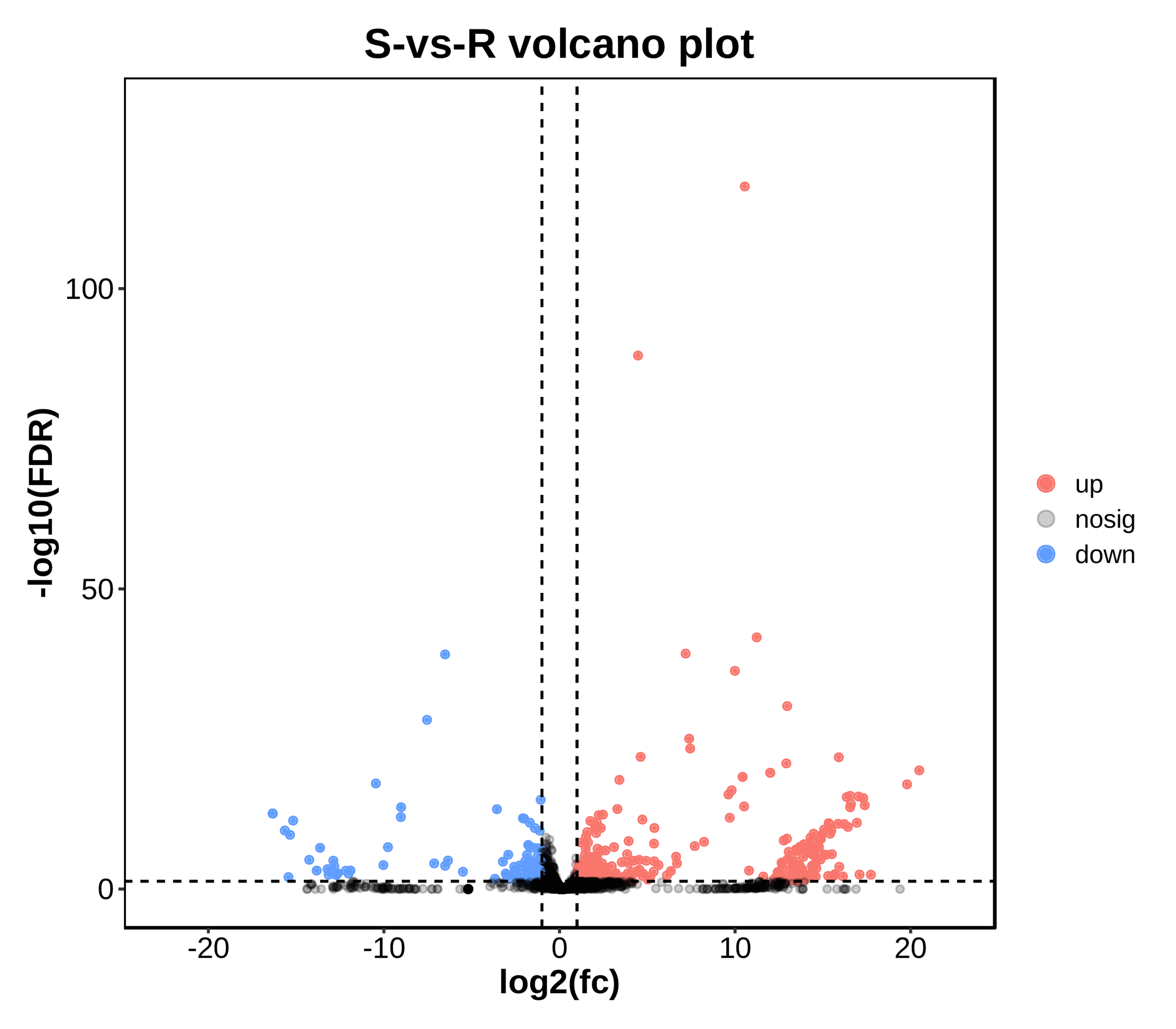

Supplement: Supplementary file 6 [file Image1.jpg]
